# Association between NT-pro-BNP levels and endothelial dysfunction assessed by digital thermal monitoring in patients with coronary artery disease

**DOI:** 10.3389/fcvm.2026.1828504

**Published:** 2026-06-12

**Authors:** Ji-Hung Wang, I-Min Su, Bang-Gee Hsu

**Affiliations:** 1 Department of Internal Medicine, Hualien Tzu Chi Hospital, Buddhist Tzu Chi Medical Foundation, Hualien, Taiwan; 2 Division of Cardiology, Hualien Tzu Chi Hospital, Buddhist Tzu Chi Medical Foundation, Hualien, Taiwan; 3 School of Medicine, Tzu Chi University, Hualien, Taiwan; 4 Department of Anesthesiology, Dalin Tzu Chi Hospital, Buddhist Tzu Chi Medical Foundation, Chiayi, Taiwan; 5 Institute of Medical Sciences, Tzu Chi University, Hualien, Taiwan; 6 Division of Nephrology, Hualien Tzu Chi Hospital, Buddhist Tzu Chi Medical Foundation, Hualien, Taiwan

**Keywords:** coronary artery disease, digital thermal monitoring, endothelial dysfunction, N-terminal pro-B-type natriuretic peptide, vascular reactivity index

## Abstract

**Objective:**

Endothelial dysfunction is a key mechanism in the progression of coronary artery disease (CAD). N-terminal pro-B-type natriuretic peptide (NT-pro-BNP), a biomarker of myocardial wall stress, has been associated with vascular abnormalities beyond overt heart failure. However, its relationship with endothelial function, as assessed via digital thermal monitoring (DTM), in patients with CAD remains unclear.

**Methods:**

We conducted a cross-sectional study including 136 patients with angiographically confirmed CAD, in whom endothelial function was evaluated using the DTM-derived vascular reactivity index (VRI). VRI values of >2.0, 1.0–1.9, and <1.0 indicated good, intermediate, and poor vascular reactivity, respectively. A commercially available electrochemiluminescence immunoassay kit was used to examine serum NT-pro-BNP levels.

**Results:**

Vascular reactivity categorized based on DTM-derived VRI was poor in 8.8% (*n* = 12) of patients, intermediate in 49.3% (*n* = 67), and good in 41.9% (*n* = 57). Poor reactivity was associated with old age (*p* = 0.031); high levels of serum total cholesterol (*p* = 0.004), low-density lipoprotein cholesterol (*p* = 0.029), and NT-pro-BNP (*p* < 0.001); and low serum albumin levels (*p* = 0.023). Higher NT-pro-BNP was independently associated with vascular reactivity dysfunction (combined intermediate and poor groups) after adjustment for covariates; each 1-standard deviation increase in log-transformed NT-pro-BNP was associated with a higher likelihood of vascular reactivity dysfunction (*β* = 0.818, 95% confidence intervals: 0.175–1.461, *p* = 0.013). This association was consistently observed across penalized logistic regression models, including LASSO, Ridge, and Elastic Net regression. In the multivariable forward stepwise linear regression analysis, the log-transformed NT-pro-BNP level remained independently and inversely associated with VRI (*p* < 0.001).

**Conclusions:**

In patients with CAD, high serum NT-pro-BNP levels were independently correlated with reduced VRI. Hence, NT-pro-BNP is independently associated with vascular reactivity dysfunction.

## 1 Introduction

Coronary artery disease (CAD) remains a major cause of morbidity and mortality worldwide and is increasingly recognized as a systemic vascular disorder rather than a disease limited to epicardial coronary stenosis. Endothelial dysfunction plays a central role in the initiation and progression of atherosclerosis by promoting inflammation, oxidative stress, vasomotor imbalance, and thrombogenicity, thereby contributing to plaque development and vascular instability (1–3). Because endothelial dysfunction can occur early in CAD and is associated with adverse cardiovascular outcomes, its assessment may provide clinically relevant information regarding vascular injury and cardiovascular risk (4–7).

Digital thermal monitoring (DTM) is a noninvasive method for evaluating peripheral vascular reactivity by measuring fingertip temperature changes during reactive hyperemia. The DTM-derived vascular reactivity index (VRI) reflects microvascular and endothelial function and has been associated with cardiovascular risk factors and vascular dysfunction in several clinical populations (8–11). In patients with CAD, DTM-derived VRI has also been applied to assess vascular reactivity and has shown associations with clinically relevant vascular and metabolic biomarkers, supporting its feasibility in this population (12).

N-terminal pro-B-type natriuretic peptide (NT-pro-BNP) is a well-established biomarker released in response to myocardial wall stress and is widely used for the diagnosis and risk stratification of heart failure and cardiac dysfunction (13–15). Beyond its cardiac implications, increasing evidence suggests that NT-pro-BNP may also reflect systemic vascular abnormalities, including microvascular damage and endothelial dysfunction (16). In patients with coronary microvascular dysfunction, elevated NT-pro-BNP levels have been observed even in the setting of preserved left ventricular function, suggesting that NT-pro-BNP may capture vascular abnormalities beyond overt myocardial impairment (17). However, the relationship between NT-pro-BNP levels and endothelial dysfunction assessed using DTM-derived VRI in patients with angiographically confirmed CAD remains insufficiently investigated. Therefore, this study aimed to evaluate the association between serum NT-pro-BNP levels and vascular reactivity assessed by DTM in patients with CAD.

## 2 Materials and methods

### 2.1 Study population

Patients diagnosed with CAD who visited the cardiovascular outpatient clinic of Hualien Tzu Chi Medical Center were consecutively screened for inclusion in this cross-sectional study. In total, 136 participants with angiographically confirmed CAD were enrolled between January 2021 and July 2021. CAD was defined as ≥50% luminal narrowing in at least one major epicardial coronary artery, as assessed by physician visual estimation during coronary angiography. Data on clinical characteristics, comorbidities, and medication exposure were collected via a detailed medical record review. All included patients were clinically stable at the time of evaluation. Patients with a history of heart failure were classified as New York Heart Association functional class I.

According to the Eighth Joint National Committee criteria, hypertension was defined as a systolic blood pressure ≥140 mmHg, a diastolic blood pressure ≥90 mmHg, or ongoing antihypertensive therapy (18). Diabetes mellitus was defined as a fasting plasma glucose level ≥126 mg/dL, a documented clinical diagnosis of diabetes mellitus in the medical records, or current use of glucose-lowering medications.

The exclusion criteria included patients with acute systemic illness or unstable cardiovascular conditions at the time of evaluation, including acute infection, acute coronary syndrome, malignancy, liver cirrhosis, uncontrolled arrhythmia, limb amputation, chronic obstructive pulmonary disease, emphysema, those who were receiving treatment of immunosuppressive agents, hospitalization or emergency treatment for heart failure within the preceding 3 months, and those who did not provide informed consent. All participants provided a written informed consent prior to study participation. The Research Ethics Committee of Hualien Tzu Chi Hospital, Buddhist Tzu Chi Medical Foundation, approved the study protocol (IRB no. 108-219-A; approval date: November 19, 2019).

### 2.2 Clinical and laboratory assessment

All examinations were performed after an overnight fast of 8–12 h. Anthropometric measurements were obtained using standardized procedures, and body mass index (BMI) was calculated as weight divided by height in meters squared (kg/m^2^). Blood pressure was measured in the morning after sufficient rest, and the mean of two readings taken 5 min apart was used for subsequent analyses.

A 5-mL venous blood sample was obtained after a fasting period of 8–12 h. A part of each sample was used for hemoglobin analysis (Sysmex XS-1000i, Sysmex America, Mundelein, IL, the USA). Other samples were collected for routine biochemical analyses, including triglyceride, total cholesterol (TCH), low-density lipoprotein cholesterol (LDL-C), high-density lipoprotein cholesterol (HDL-C), fasting glucose, albumin, blood urea nitrogen (BUN), and creatinine levels, using an automated analyzer (Advia 1800; Siemens Healthcare GmbH, Henkestr, Germany). Circulating NT-pro-BNP concentrations were quantified using an electrochemiluminescence immunoassay (Elecsys 2010 Immunoanalyzer; Roche Diagnostics, Indianapolis, IN, USA). Renal function, as estimated glomerular filtration rate (eGFR), was assessed using the 2021 Chronic Kidney Disease Epidemiology Collaboration equation.

### 2.3 Assessment of endothelial function

Peripheral vascular function was evaluated using DTM (Endothelix Inc., Houston, TX, USA), which assesses temperature rebound during reactive hyperemia as an index of vascular reactivity. The participants did not smoke or consume alcoholic drinks, caffeinated beverages, and vasoactive medications prior to testing. Measurements were conducted in a temperature-controlled laboratory environment (approximately 24 °C) after a resting period of at least 15 min while in the supine position.

Temperature sensors were applied to both index fingers, with one side serving as a reference. After stabilization, arterial occlusion was induced by inflating the upper arm cuff to 50 mmHg above the systolic blood pressure for 5 min. Subsequent cuff release triggered reactive hyperemia, and fingertip temperature changes were continuously recorded. The vascular reactivity index (VRI) was automatically calculated using the VENDYS-II software based on the maximal temperature difference between the baseline and recovery phases. VRI values were categorized as good (≥2.0), intermediate (1.0–1.9), or poor (<1.0) vascular reactivity. In the present study, vascular reactivity dysfunction was defined as intermediate or poor vascular reactivity (VRI < 2.0), whereas good vascular reactivity (VRI ≥ 2.0) was considered preserved vascular reactivity (19).

### 2.4 Transthoracic echocardiography

Transthoracic echocardiography was performed using a Sonos 5500 Cardiovascular Ultrasound system (Hewlett-Packard/Philips, Andover, MA, USA). Echocardiographic parameters collected for analysis included left ventricular ejection fraction (LVEF), left atrial (LA) diameter, transmitral E/A ratio, average E/e′ ratio, LA volume index (LAVI), the presence of left ventricular diastolic dysfunction, and LA enlargement. LV diastolic dysfunction was assessed by integrated echocardiographic evaluation of LV relaxation and filling pressure, using available parameters, including the E/A ratio, the average E/e′ ratio, and the LAVI, according to the updated recommendations of the American Society of Echocardiography (20). LA enlargement was defined by an LAVI >34 mL/m^2^.

### 2.5 Statistical analysis

Analyses were performed with consideration of the distributional characteristics of individual variables. Continuous data were reported as mean ± standard deviation or median with interquartile range, as appropriate. Meanwhile, categorical variables were summarized as frequencies and proportions. Differences among vascular reactivity categories were evaluated for trend using one-way analysis of variance for variables with a normal distribution and the Jonckheere–Terpstra test for skewed data. Categorical variables were compared using the Cochran–Armitage test.

Several biochemical parameters (such as triglyceride, TCH, LDL-C, HDL-C, fasting glucose, BUN, creatinine, E/A ratio, LAVI, and NT-pro-BNP levels) had skewed distributions. Thus, a logarithmic transformation was applied before regression analyses. The association between VRI and clinical variables was initially explored using a simple linear regression model. To identify the independent determinants of VRI, variables showing significant associations were subsequently entered into a multivariate stepwise linear and forced-entry regression model. Multicollinearity was assessed using variance inflation factors.

Multivariable logistic regression was performed to evaluate factors associated with vascular reactivity dysfunction (intermediate or poor vascular reactivity), including age, log-TCH, log-LDL-C, albumin, eGFR, LVEF, log-LAVI, and log-NT-pro-BNP as covariates (*p* < 0.2 in trend differences across vascular reactivity categories were considered for multivariable analysis). Regression results were reported as *β* coefficients with 95% confidence intervals (CI) and corresponding *p*-values. To further assess model robustness and reduce potential overfitting, penalized logistic regression analyses were additionally performed using Least Absolute Shrinkage and Selection Operator (LASSO), Ridge, and Elastic Net regression with the same covariates. Continuous predictors were standardized before both multivariable logistic regression and penalized logistic regression analyses so that *β* coefficients represented changes in log odds per 1-standard deviation increase in each continuous predictor. For penalized models, *β* coefficients, 95% CI, and *p*-values were estimated using resampling-based methods because conventional standard errors are not directly available from penalized regression models.

The discriminatory ability of NT-pro-BNP for vascular reactivity dysfunction (intermediate or poor vascular reactivity) was evaluated using receiver operating characteristic curve (ROC) analysis. The area under the ROC curve was calculated with 95% CI, and the optimal cutoff value was determined using the Youden index. Sensitivity and specificity were calculated at the optimal cutoff. Calibration was assessed using the Brier score, Hosmer–Lemeshow goodness-of-fit test, calibration intercept, calibration slope, and calibration plot. The calibration plot was visually inspected to evaluate agreement between predicted and observed probabilities across predicted-risk groups. To reduce optimism bias and internally validate model performance, bootstrap resampling was performed to estimate optimism-corrected AUC, Brier score, calibration intercept, and calibration slope. Decision curve analysis (DCA) was performed to evaluate the clinical utility of the NT-pro-BNP-based model across a range of threshold probabilities. All analyses were conducted using the Statistical Package for the Social Sciences software (version 25.0; IBM Corp., Armonk, NY, USA) and R software (version 4.2.2).

## 3 Results

### 3.1 Clinical characteristics according to vascular reactivity

The analysis included 136 patients with angiographically confirmed CAD. Based on the VRI values obtained via DTM, 57 (41.9%) patients were classified as having good vascular reactivity, 67 (49.3%) as having intermediate vascular reactivity, and 12 (8.8%) as having poor vascular reactivity.

Table 1 presents the baseline characteristics across vascular reactivity categories. Patients with poor vascular reactivity were older (*p* = 0.031) and had high serum TCH (*p* = 0.004) and LDL-C (*p* = 0.029) levels. Patients with impaired vascular reactivity had significantly low serum albumin levels (*p* for trend = 0.023). Notably, the NT-pro-BNP levels (*p* for trend <0.001) progressively increased across worsening vascular reactivity categories, and they were significantly elevated in patients with poor vascular reactivity. The other clinical parameters, including BMI, blood pressure, renal function, and prevalence of diabetes mellitus or hypertension, did not differ significantly among the good, intermediate, or poor vascular reactivity groups. There were no statistically significant trends across worsening vascular reactivity categories for left ventricular ejection fraction (LVEF), left atrial diameter, E/A ratio, average E/e′ ratio, left atrial volume index, left ventricular diastolic dysfunction, left atrial enlargement, CAD-related variables, including left main disease, prior coronary artery bypass grafting (CABG), prior percutaneous coronary intervention (PCI), and the number of diseased vessels.

**Table 1:** Clinical characteristics according to different vascular reactivity index by digital thermal monitoring of the 136 coronary artery disease patients.

Characteristics	All participants (*n* = 136)	Good vascular reactivity (*n* = 57)	Intermediate vascular reactivity (*n* = 67)	Poor vascular reactivity (*n* = 12)	*p* trend
Basic profiles					
Age (years)	62.80 ± 8.82	61.19 ± 8.62	63.38 ± 8.81	67.24 ± 8.54	0.031*
Body mass index (kg/m^2^)	26.49 ± 3.62	26.61 ± 3.56	26.53 ± 3.79	25.70 ± 3.11	0.434
Vascular reactivity index	1.85 ± 0.62	2.40 ± 0.32	1.63 ± 0.25	0.52 ± 0.23	<0.001*
Systolic blood pressure (mmHg)	132.13 ± 17.90	134.02 ± 16.60	130.36 ± 17.88	133.00 ± 23.94	0.809
Diastolic blood pressure (mmHg)	77.59 ± 12.80	78.40 ± 14.77	77.57 ± 11.11	73.83 ± 11.94	0.264
Male, *n* (%)	117 (86.7)	46 (80.7)	60 (90.9)	11 (91.7)	0.218
Smoking, *n* (%)	32 (23.5)	15 (26.3)	14 (20.9)	3 (25.0)	0.772
CAD profiles					
Left main, *n* (%)	57 (41.9)	27 (47.4)	25 (37.3)	5 (41.7)	0.527
CABG, *n* (%)	17 (12.5)	5 (8.8)	10 (14.9)	2 (16.7)	0.518
Prior PCI, *n* (%)	34 (25.0)	13 (27.8)	16 (23.9)	5 (41.7)	0.410
Single vessel disease, *n* (%)	71 (52.2)	31 (54.4)	35 (52.2)	5 (41.7)	0.575
Double vessel disease, *n* (%)	28 (20.6)	14 (24.6)	12 (17.9)	2 (16.7)	
Triple vessel disease, *n* (%)	37 (27.2)	12 (21.1)	20 (29.9)	5 (41.7)	
Laboratory data					
Hemoglobin (g/dL)	14.20 ± 1.76	13.86 ± 1.72	14.60 ± 1.57	13.55 ± 2.49	0.571
Total cholesterol (mg/dL)	160.00 (139.00–178.75)	154.00 (140.00–174.00)	154.00 (136.00–180.00)	178.00 (171.50–217.75)	0.004*
Triglyceride (mg/dL)	131.50 (98.25–183.75)	131.00 (100.00–194.00)	131.00 (91.00–181.00)	157.50 (109.25–207.50)	0.587
HDL-C (mg/dL)	43.00 (38.00–53.00)	45.00 (38.00–55.00)	42.00 (38.00–53.00)	46.00 (38.00–50.75)	0.671
LDL-C (mg/dL)	92.00 (78.25–112.75)	89.00 (74.00–103.50)	92.00 (80.00–129.00)	106.00 (91.25–140.25)	0.029*
Fasting glucose (mg/dL)	107.50 (92.00–141.00)	109.00 (91.50–143.50)	110.00 (92.00–146.00)	103.00 (82.00–125.25)	0.472
Albumin (g/dL)	4.39 ± 0.23	4.42 ± 0.26	4.38 ± 0.19	4.26 ± 0.27	0.023*
Blood urea nitrogen (mg/dL)	16.00 (13.00–19.75)	16.00 (13.00–18.00)	17.00 (14.00–21.00)	17.00 (13.25–24.25)	0.412
Creatinine (mg/dL)	1.00 (0.80–1.10)	1.00 (0.80–1.10)	1.00 (0.80–1.10)	1.00 (0.90–1.35)	0.465
eGFR (mL/min)	82.60 ± 23.57	84.80 ± 25.77	82.22 ± 21.72	74.30 ± 22.46	0.163
NT-pro-BNP (pg/mL)	155.65 (71.79–348.93)	96.53 (48.24–156.65)	187.11 (73.36–376.68)	1,448.11 (692.64–2,192.31)	<0.001*
Underlying diseases					
Diabetes mellitus, *n* (%)	62 (45.6)	28 (49.1)	29 (43.3)	5 (41.7)	0.777
Hypertension, *n* (%)	78 (57.4)	35 (61.4)	37 (55.2)	6 (50.0)	0.680
Heart failure history, *n* (%)	33 (24.6)	14 (25.0)	15 (22.7)	4 (33.3)	0.744
Medical therapy					
ACEi/ARB use, *n* (%)	82 (60.3)	38 (66.7)	39 (58.2)	5 (41.7)	0.243
*β*-blocker use, *n* (%)	73 (53.7)	30 (52.6)	36 (53.7)	7 (58.3)	0.937
CCB use, *n* (%)	53 (39.0)	25 (43.9)	22 (32.8)	6 (50.0)	0.325
Statin use, *n* (%)	113 (83.1)	46 (80.7)	57 (85.1)	10 (83.3)	0.811
Fibrate use, *n* (%)	17 (12.5)	9 (15.8)	6 (9.0)	2 (16.7)	0.467
Aspirin, *n* (%)	101 (74.3)	43 (75.4)	49 (73.1)	9 (75.0)	0.956
Clopidogrel, *n* (%)	49 (36.0)	21 (36.8)	23 (34.3)	5 (41.7)	0.877
Echocardiographic parameters					
LV ejection fraction (%)	66.26 ± 12.44	67.71 ± 12.72	66.24 ± 11.97	60.44 ± 13.41	0.099
LA diameter (cm)	3.57 ± 0.57	3.73 ± 0.61	3.47 ± 0.54	3.51 ± 0.49	0.304
E/A ratio	0.80 (0.70–1.00)	0.82 (0.73–1.30)	0.79 (0.71–0.93)	0.85 (0.57–1.07)	0.345
Average E/e′ ratio	12.37 ± 4.87	12.58 ± 6.88	12.14 ± 3.78	12.99 ± 3.55	0.854
LA volume index (mL/m^2^)	31.50 (30.00–33.30)	32.00 (31.00–35.00)	31.00 (29.90–32.75)	31.50 (30.05–36.00)	0.106
LV diastolic dysfunction, *n* (%)	38 (27.9)	12 (21.1)	21 (31.3)	5 (41.7)	0.242
LA enlargement, *n* (%)	21 (15.4)	12 (21.1)	7 (10.4)	2 (16.7)	0.261

Measured values between groups (poor, intermediate, and good VRI) were assessed using a Jonckheere–Terpstra test for parameters with non-normal distribution, and one-way analysis of variance for normally distributed data, and categorical variables were compared using the Cochran–Armitage test for trend. CAD, coronary artery disease; CABG, coronary artery bypass grafting; PCI, percutaneous coronary intervention; HDL-C, high-density lipoprotein cholesterol; LDL-C, low-density lipoprotein cholesterol; eGFR, estimated glomerular filtration rate; NT-pro-BNP, N-terminal pro-B-type natriuretic peptide; ACEi, angiotensin-converting enzyme inhibitor; ARB, angiotensin-receptor blocker; CCB, calcium-channel blocker; LV, left ventricular; LA, left atrium.

* *p* < 0.05 was considered statistically significant.

### 3.2 Association between clinical variables and VRI

The associations between clinical variables and VRI were further evaluated using linear regression analyses (Table 2). In the simple linear regression analyses, age (*r* = −0.200, *p* = 0.019), log-transformed TCH levels (log-TCH, *r* = −0.196, *p* = 0.022), log-LDL-C levels (*r* = −0.266, *p* = 0.002), albumin levels (*r* = 0.185, *p* = 0.031), and log-NT-pro-BNP levels (*r* = −0.469, *p* < 0.001) were significantly associated with VRI values. LVEF, left atrial diameter, E/A ratio, average E/e′ ratio, log-LAVI were not significantly associated with VRI in simple linear regression analysis. In the stepwise multivariable linear regression model, log-NT-pro-BNP was the only retained independent factor associated with VRI, explaining 22.0% of the variance in VRI, with an adjusted *R*^2^ of 0.214. Higher log-NT-pro-BNP was significantly associated with lower VRI values (*B* = −0.579, standardized *β* = −0.469, *p* < 0.001). To address the potential instability of stepwise variable selection, a forced-entry multivariable linear regression model was additionally performed using age, albumin, log-LDL-C, log-TCH, and log-NT-pro-BNP. The forced-entry model was statistically significant and explained 28.4% of the variance in VRI, with an adjusted *R*^2^ of 0.257 (*p* < 0.001). In this model, log-NT-pro-BNP remained independently and inversely associated with VRI (*B* = −0.466, standardized *β* = −0.377, *p* < 0.001), whereas age, albumin, log-LDL-C, and log-TCH were not independently associated with VRI after adjustment. Variance inflation factor values ranged from 1.061 to 1.235, indicating no substantial multicollinearity among the included covariates.

**Table 2:** Correlation of vascular reactivity index levels and clinical variables by simple or multivariable linear regression analyses among 136 coronary artery disease patients.

Variables	Vascular reactivity index								
			Multivariable regression						
	Simple regression		Stepwise model			Forced-entry model			
	*r*	*p* value	*B*	*β*	*p* value	*B*	*β*	*p* value	VIF
Age (years)	−0.200	0.019*	–	–	–	−0.008	−0.119	0.122	1.061
Body mass index (kg/m^2^)	−0.008	0.931	–	–	–	–	–	–	–
Systolic blood pressure (mmHg)	0.093	0.284	–	–	–	–	–	–	–
Diastolic blood pressure (mmHg)	0.140	0.103	–	–	–	–	–	–	–
Hemoglobin (g/dL)	−0.058	0.500	–	–	–	–	–	–	–
Log-TCH (mg/dL)	−0.196	0.022*	–	–	–	−0.695	−0.109	0.160	1.082
Log-triglyceride (mg/dL)	−0.048	0.579	–	–	–	–	–	–	–
Log-HDL-C (mg/dL)	0.078	0.366	–	–	–	–	–	–	–
Log-LDL-C (mg/dL)	−0.266	0.002*	–	–	–	−0.584	−0.138	0.097	1.235
Log-glucose (mg/dL)	0.160	0.063	–	–	–	–	–	–	–
Albumin (g/dL)	0.185	0.031*	–	–	–	0.372	0.139	0.076	1.094
Log-BUN (mg/dL)	−0.056	0.521	–	–	–	–	–	–	–
Log-creatinine (mg/dL)	−0.147	0.088	–	–	–	–	–	–	–
eGFR (mL/min)	0.138	0.110	–	–	–	–	–	–	–
Log-NT-pro-BNP (pg/mL)	−0.469	<0.001*	−0.579	−0.469	<0.001*	−0.466	−0.377	<0.001	1.170
LV ejection fraction (%)	0.100	0.309	–	–	–	–	–	–	–
LA diameter (cm)	0.071	0.520	–	–	–	–	–	–	–
Log-E/A ratio	0.089	0.421	–	–	–	–	–	–	–
Average E/e′ ratio	0.015	0.908	–	–	–	–	–	–	–
Log-LAVI (mL/m^2^)	0.192	0.100	–	–	–	–	–	–	–

Data for TCH, triglycerides, HDL-C, LDL-C, fasting glucose, BUN, creatinine, E/A ratio, LAVI, and NT-pro-BNP were log-transformed before analysis due to skewed distributions. Simple linear regression or multivariable stepwise or forced-entry linear regression was used to analyze the data (adapted factors included age, log-TCH, log-LDL-C, albumin, and log-NT-pro-BNP). *B* indicates the unstandardized regression coefficient, and standardized *β* indicates the standardized regression coefficient. Variables not retained in the stepwise model or not included in the forced-entry model are indicated by em dashes. TCH, total cholesterol; HDL-C, high-density lipoprotein cholesterol; LDL-C, low-density lipoprotein cholesterol; BUN, blood urea nitrogen; eGFR, estimated glomerular filtration rate; NT-pro-BNP, N-terminal pro-B-type natriuretic peptide; LV, left ventricular; LA, left atrial; LAVI, left atrial volume index; VIF, variance inflation factor.

* Statistical significance was defined as *p* < 0.05. Stepwise model: *R*^2^ = 0.220, adjusted *R*^2^ = 0.214, *p* < 0.001. Forced-entry model: *R*^2^ = 0.284, adjusted *R*^2^ = 0.257, *p* < 0.001.

### 3.3 Association between NT-pro-BNP levels and vascular reactivity dysfunction

Multivariable logistic regression analysis was performed to evaluate factors associated with vascular reactivity dysfunction, defined as intermediate or poor vascular reactivity. Age, log-TCH, log-LDL-C, albumin, eGFR, LVEF, log-LAVI, and log-NT-pro-BNP were included as covariates because they showed a trend toward differences across vascular reactivity categories and were considered for multivariable analysis using a *p* < 0.20 threshold. In multivariable logistic regression using standardized predictors, log-transformed NT-pro-BNP was independently associated with vascular reactivity dysfunction after adjustment for age, log-TCH, log-LDL-C, albumin, eGFR, LVEF, and log-LAVI. Each 1-standard deviation increase in log-NT-pro-BNP was associated with a higher likelihood of vascular reactivity dysfunction (*β* = 0.818, 95% CI: 0.175–1.461, *p* = 0.013). In penalized logistic regression analyses, the positive association between log-NT-pro-BNP and vascular reactivity dysfunction remained consistent across LASSO (*β* = 0.645, 95% CI: 0.182–1.256, *p* = 0.010), Ridge (*β* = 0.419, 95% CI: 0.212–0.637, *p* = 0.002), and Elastic Net models (*β* = 0.469, 95% CI: 0.209–0.744, *p* = 0.002), supporting the robustness of this association (Table 3).

**Table 3:** Multivariable and penalized logistic regression analyses of factors associated with vascular reactivity dysfunction.

Factors	Multivariable		LASSO		Ridge		Elastic net	
	*β* (95% CI)	*p* value	*β* (95% CI)	*p* value	*β* (95% CI)	*p* value	*β* (95% CI)	*p* value
log-NT-pro-BNP	0.818 (0.175, 1.461)	0.013*	0.645 (0.182, 1.256)	0.010*	0.419 (0.212, 0.637)	0.002*	0.469 (0.209, 0.744)	0.002*
Age	−0.037 (−0.582, 0.508)	0.894	0.000 (−0.305, 0.389)	1.000	0.023 (−0.234, 0.287)	0.933	0.000 (−0.246, 0.308)	1.000
log-TCH	−0.396 (−1.295, 0.504)	0.388	−0.026 (−0.386, 0.000)	1.000	−0.105 (−0.286, 0.125)	0.366	−0.088 (−0.307, 0.092)	0.557
log-LDL-C	0.617 (−0.213, 1.447)	0.145	0.322 (0.000, 0.883)	0.292	0.291 (0.060, 0.509)	0.016*	0.310 (0.020, 0.581)	0.044*
Albumin	−0.185 (−0.752, 0.381)	0.521	−0.050 (−0.549, 0.114)	1.000	−0.139 (−0.371, 0.135)	0.328	−0.129 (−0.409, 0.130)	0.547
eGFR	0.017 (−0.519, 0.553)	0.949	0.000 (−0.308, 0.387)	1.000	0.037 (−0.232, 0.296)	0.721	0.001 (−0.242, 0.315)	0.935
LVEF	−0.114 (−0.694, 0.466)	0.701	0.000 (−0.487, 0.176)	1.000	−0.046 (−0.334, 0.189)	0.631	−0.018 (−0.364, 0.184)	0.849
log-LAVI	−0.515 (−1.055, 0.024)	0.061	−0.297 (−0.845, 0.000)	0.214	−0.263 (−0.519, −0.007)	0.048*	−0.272 (−0.590, 0.000)	0.080

Values are presented as *β* coefficients with 95% confidence intervals per 1-standard deviation increase in each continuous predictor. Vascular reactivity dysfunction was defined as intermediate or poor vascular reactivity vs. good vascular reactivity. TCH, LDL-C, LAVI, and NT-pro-BNP were log-transformed before standardization. The multivariable logistic regression model included age, TCH, LDL-C, albumin, eGFR, LVEF, LAVI, and NT-pro-BNP as covariates. Penalized logistic regression models were fitted using standardized predictors with 5-fold cross-validation for tuning. Confidence intervals and *p*-values for penalized models were estimated using stratified bootstrap resampling with 1,000 iterations. CI, confidence interval; LASSO, least absolute shrinkage and selection operator; TCH, total cholesterol; LDL-C, low-density lipoprotein cholesterol; eGFR, estimated glomerular filtration rate; LVEF, left ventricular ejection fraction; LAVI, left atrial volume index; NT-pro-BNP, N-terminal pro-B-type natriuretic peptide.

* *p* < 0.05 was considered statistically significant.

### 3.4 Discrimination, calibration, and clinical utility of NT-pro-BNP for vascular reactivity dysfunction

NT-pro-BNP demonstrated moderate discriminatory ability for identifying vascular reactivity dysfunction, defined as intermediate or poor vascular reactivity. Among 136 patients with available data, 79 patients had vascular reactivity dysfunction. The area under the ROC was 0.740 (95% CI, 0.654–0.819; *p* < 0.001). The optimal NT-proBNP cutoff determined by the Youden index was 233.17 pg/mL, with a sensitivity of 57.0% and specificity of 91.2%. Calibration performance was acceptable, with a Brier score of 0.194 and a non-significant Hosmer–Lemeshow goodness-of-fit test (*p* = 0.407), indicating no evidence of poor model calibration (Table 4).

**Table 4:** Apparent and bootstrap-corrected discrimination and calibration performance of N-terminal pro-B-type natriuretic peptide for vascular reactivity dysfunction (intermediate vascular reactivity and poor vascular reactivity).

Outcome	Events/Total	AUC (95% CI) *p* value	Optimal cutoff (pg/mL)	Sensitivity	Specificity	Brier score	Hosmer-Lemeshow *p* value	Bootstrap-corrected AUC	Bootstrap-corrected Brier score	Bootstrap-corrected calibration intercept	Bootstrap-corrected calibration slope
Vascular reactivity dysfunction	79/136	0.740 (0.654, 0.819) <0.001*	233.17	0.570	0.912	0.194	0.407	0.741	0.197	0.004	1.020

Vascular reactivity dysfunction was defined as intermediate or poor vascular reactivity compared with good vascular reactivity. The analysis used complete cases with available vascular reactivity dysfunction status and NT-pro-BNP measurements. The AUC 95% CI was estimated using stratified bootstrap resampling (5,000 iterations), and the AUC *p*-value was calculated using the Mann–Whitney *U* test. The optimal cutoff was selected using the Youden index. Brier score and Hosmer–Lemeshow *p*-value were calculated from a univariable logistic regression model using NT-pro-BNP as the predictor. Internal validation was performed using stratified bootstrap resampling (5,000 iterations) to estimate optimism-corrected AUC, Brier score, calibration intercept, and calibration slope. AUC, area under the receiver operating characteristic curve; CI, confidence interval; NT-pro-BNP, N-terminal pro-B-type natriuretic peptide.

* *p* < 0.05 was considered statistically significant.

**Figure 1:**
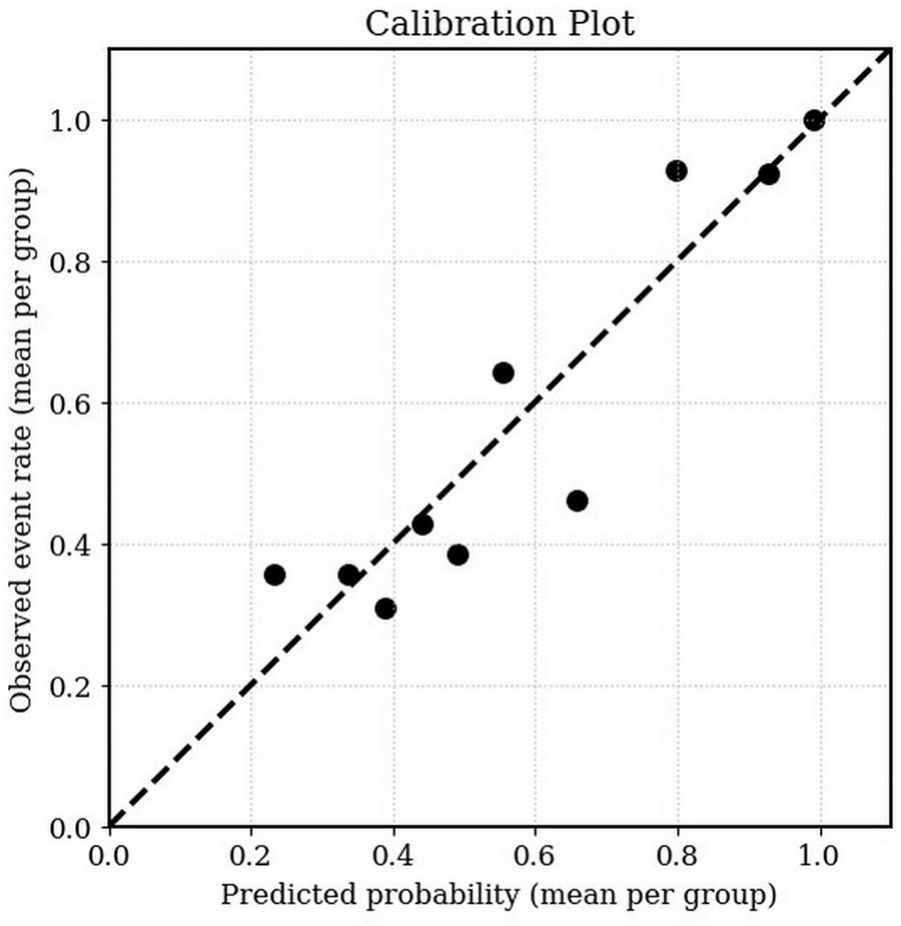
Calibration plot for vascular reactivity dysfunction.

**Figure 2:**
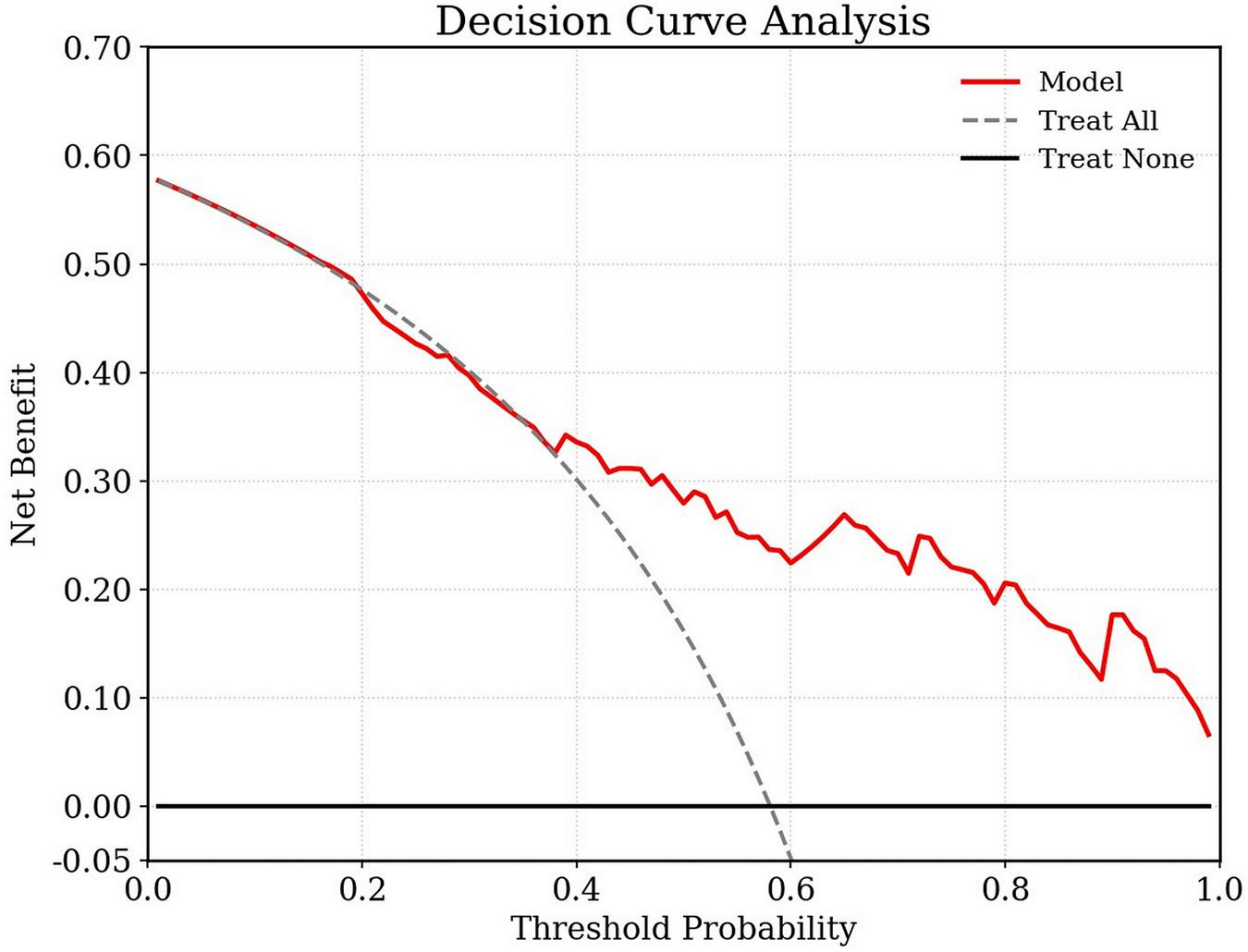
Decision curve analysis for the NT-pro-BNP-based logistic model predicting vascular reactivity dysfunction.

Calibration analysis showed an overall acceptable but imperfect agreement between predicted and observed probabilities. The calibration intercept was close to zero (0.000 [95% CI: −0.393 to 0.393]), and the calibration slope was near one (1.000 [95% CI: 0.542–1.459]) (Figure 1). However, visual inspection of the calibration plot showed non-trivial deviations from the identity line, particularly in the intermediate predicted probability range of approximately 0.4–0.7. Therefore, calibration was interpreted as acceptable overall but not uniformly optimal across the full range of predicted probabilities. Internal validation using bootstrap resampling showed similar optimism-corrected performance, with a bootstrap-corrected AUC of approximately 0.741, Brier score of approximately 0.197, calibration intercept of approximately 0.004, and calibration slope of approximately 1.020 (Table 4). DCA further showed that the NT-pro-BNP-based model provided a positive net clinical benefit across various threshold probabilities compared with default strategies (Figure 2).

## 4 Discussion

In this cross-sectional study of patients with angiographically confirmed CAD, circulating NT-pro-BNP levels were significantly associated with endothelial dysfunction assessed via DTM. High NT-pro-BNP concentrations were independently associated with lower VRI values and vascular reactivity dysfunction after multivariate adjustment. Based on these findings, NT-pro-BNP, traditionally considered a biomarker of myocardial wall stress, may also reflect systemic vascular dysfunction in patients diagnosed with CAD.

In addition to NT-pro-BNP, several clinical variables, including age, lipid parameters, and serum albumin levels, differed significantly across vascular reactivity categories. These findings are biologically plausible and consistent with established mechanisms underlying vascular dysfunction. Advancing age is strongly associated with progressive impairment of endothelial function. Aging is characterized by increased oxidative stress, reduced nitric oxide bioavailability, mitochondrial dysfunction, and chronic low-grade inflammation, all of which contribute to vascular stiffening and endothelial dysfunction (21–23). Both experimental and human studies have well documented structural and functional alterations in the aging vasculature, including impaired endothelial-dependent vasodilation and diminished reparative capacity (22, 23). Therefore, the observed association between older age and poorer vascular reactivity in our cohort is in accordance with the concept of vascular aging as a central determinant of endothelial dysfunction. Further, dyslipidemia plays a fundamental role in endothelial injury. Elevated levels of atherogenic lipoproteins promote the oxidative modification of LDL particles, endothelial activation, and inflammatory signaling, leading to impaired nitric oxide production and increased vascular permeability (24, 25). Lipid-induced endothelial dysfunction is an early step in atherogenesis and has been shown to precede overt structural plaque formation (24, 26). In our study, higher TCH and LDL-C levels were associated with impaired vascular reactivity, thereby supporting the established association between lipid abnormalities and endothelial dysfunction. Interestingly, patients with poor vascular reactivity have low serum albumin levels. Albumin exerts antioxidant and anti-inflammatory effects and contributes to the maintenance of endothelial integrity by binding reactive oxygen species and modulating endothelial permeability (27, 28). Hypoalbuminemia has been associated with adverse cardiovascular outcomes and may reflect systemic inflammation, malnutrition, or increased disease burden (27, 29). The association between low albumin levels and impaired vascular reactivity in our study indicates that diminished antioxidant capacity and heightened inflammatory stress may contribute to endothelial dysfunction in patients with CAD. Taken together, these findings reinforce the multifactorial nature of endothelial dysfunction and support the biological coherence of our results.

Beyond traditional cardiovascular risk factors, the association between NT-pro-BNP levels and endothelial dysfunction observed in our study is supported by emerging evidence linking natriuretic peptides to vascular health. Along with evidence showing functional heterogeneity among circulating natriuretic peptide forms (30), these findings indicate that NT-pro-BNP may be an integrative biomarker reflecting both cardiac and vascular pathophysiology. In a large multi-ethnic cohort, NT-pro-BNP levels were independently associated with impaired endothelial function, even after adjusting for conventional risk factors, suggesting that natriuretic peptides may reflect vascular abnormalities beyond overt heart failure (31). Similarly, clinical studies have revealed that B-type natriuretic peptide levels are independently related to endothelial function assessed by vascular reactivity testing, thereby reinforcing the concept that natriuretic peptide signaling is closely intertwined with endothelial physiology (32). In addition to peripheral endothelial measurements, elevated NT-pro-BNP levels have been associated with microvascular damage in population-based studies. For example, high NT-pro-BNP concentrations were associated with retinal microvascular abnormalities, thereby supporting the notion that NT-pro-BNP levels may reflect systemic microvascular injury rather than isolated myocardial stress (16). These findings are consistent with our observation that NT-pro-BNP is associated with impaired DTM-derived VRI in patients with CAD. Experimental data further suggest that natriuretic peptides may exert direct effects on endothelial biology. B-type natriuretic peptide enhances vasculogenesis by promoting the number and functional capacity of endothelial progenitor cells (33). This dual role reflecting hemodynamic stress while potentially regulating endothelial repair mechanisms emphasizes the complex interaction between natriuretic peptide signaling and vascular homeostasis. Taken together, these clinical and experimental findings provide mechanistic plausibility for the observed association between NT-pro-BNP levels and endothelial dysfunction in our study. Importantly, NT-pro-BNP is influenced by myocardial wall stress and may reflect subclinical systolic or diastolic dysfunction, even in patients without decompensated heart failure. Therefore, the association between NT-pro-BNP and DTM-derived VRI should not be interpreted as evidence of a direct causal effect of natriuretic peptide signaling on endothelial dysfunction. Rather, NT-pro-BNP may serve as an integrative biomarker linking cardiac load, compensated cardiac dysfunction, and systemic vascular impairment in patients with CAD. However, considering the cross-sectional nature of this study, it remains unclear whether elevated NT-pro-BNP levels contribute to endothelial dysfunction, are caused by shared upstream pathophysiological processes, or represent a compensatory response to vascular impairment.

Beyond demonstrating a significant association between NT-pro-BNP levels and endothelial dysfunction, our study further explored its potential clinical utility for identifying impaired vascular reactivity in patients with CAD. In the ROC analysis, NT-pro-BNP showed moderate discriminative performance for vascular reactivity dysfunction, defined as intermediate or poor vascular reactivity compared with good vascular reactivity. Because only 12 patients were classified as having poor vascular reactivity, we did not emphasize poor vascular reactivity as a separate primary outcome in the predictive analysis, thereby reducing the risk of unstable estimates and overfitting caused by the limited number of events. To provide a more comprehensive evaluation of model performance, we further assessed calibration and clinical utility. Calibration analysis suggested generally acceptable agreement between predicted and observed probabilities; however, the relatively wide confidence intervals for the calibration intercept and slope, together with visible deviations from the ideal line in the intermediate predicted probability range, indicate that the model may not provide uniformly precise risk estimates across all risk strata. Therefore, the calibration findings should be interpreted as exploratory and require confirmation in larger cohorts with internal and external validation. In addition, DCA showed that the NT-pro-BNP-based model provided a positive net benefit across a range of threshold probabilities compared with default “treat-all” or “treat-none” strategies, suggesting potential clinical usefulness. From a clinical perspective, these findings suggest that NT-pro-BNP may provide information beyond myocardial wall stress in patients with CAD. In line with current guidelines emphasizing comprehensive risk assessment, cardiac functional evaluation, and individualized long-term management in patients with chronic coronary syndromes (34), elevated NT-pro-BNP levels in clinically stable CAD patients may prompt clinicians to consider a broader vascular risk profile, including possible endothelial or microvascular dysfunction. However, NT-pro-BNP should not be interpreted as a standalone diagnostic marker of endothelial dysfunction. Rather, it may serve as a complementary biomarker to help identify patients who could benefit from closer cardiovascular risk assessment, optimization of guideline-directed medical therapy, and further evaluation of vascular function when clinically appropriate.

The current study has several strengths that should be considered. First, endothelial function was assessed using DTM, a noninvasive and operator-independent method that reflects microvascular reactivity and allows the standardized evaluation of vascular function in clinical settings. Second, both continuous (VRI) and categorical (endothelial dysfunction) outcomes were examined, thereby enabling the assessment of physiological associations and clinically relevant classification. Third, beyond conventional discrimination analysis, we incorporated calibration assessment and DCA, providing a more comprehensive evaluation of model performance and potential clinical utility. Nevertheless, several limitations of this study should be acknowledged. First, because this was a retrospective, cross-sectional observational study, causal inference cannot be established, and the temporal relationship between elevated NT-pro-BNP levels and endothelial dysfunction remains unclear. Although multivariable adjustment was performed, residual confounding from unmeasured or incompletely characterized factors remains possible. In particular, NT-pro-BNP levels may be influenced by renal function, cardiac functional status, ischemic burden, and other hemodynamic conditions. Although eGFR and available echocardiographic parameters were included in the clinical assessment, and patients with hospitalization or emergency treatment for heart failure within the preceding 3 months were excluded, subtle interactions among cardiac, renal, and vascular physiology may not have been fully captured. Compensated cardiac dysfunction or heart failure with preserved ejection fraction may still have been present in some patients; therefore, residual confounding related to cardiac functional status cannot be fully excluded. In addition, although CAD-related variables, including left main disease, prior PCI, prior CABG, and the number of diseased vessels, were reported, more detailed anatomical assessments of CAD complexity, such as the SYNTAX score, were not routinely available. Coronary stenosis was assessed by physician visual estimation rather than quantitative coronary angiography; therefore, potential inter-observer variability, particularly for lesions near the 50% stenosis threshold, cannot be excluded. Residual confounding related to coronary lesion complexity, ischemic burden, or completeness of revascularization may also remain. Second, the sample size was determined by the number of eligible patients with available VRI and NT-pro-BNP data during the study period, and no formal *a priori* sample size calculation was performed. To address this issue, a *post hoc* sensitivity power analysis was performed for the primary linear regression analysis using VRI as a continuous outcome. With 136 patients, five predictors in the forced-entry multivariable linear regression model, a two-sided alpha level of 0.05, and 80% power, the study was able to detect an overall effect size of approximately *f*^2^ = 0.10. The observed overall effect size of the forced-entry model was *f*^2^ = 0.397, calculated from the model *R*^2^ of 0.284. However, the number of patients with poor vascular reactivity was small, and analyses focusing specifically on this subgroup may be statistically unstable. Third, although DTM provides a noninvasive and operator-independent assessment of vascular reactivity, the VRI cutoff values were adopted from previously reported DTM-based classifications rather than being specifically derived for the present CAD cohort. Moreover, validation of DTM against flow-mediated dilation has been performed mainly in selected populations; therefore, further studies are needed to confirm the optimal VRI thresholds and prognostic significance of DTM-derived vascular reactivity categories in patients with CAD. Finally, the study population was predominantly male, reflecting the sex distribution of the enrolled CAD cohort. Because endothelial function and NT-pro-BNP levels may differ by sex, this imbalance may limit the generalizability of the findings to female patients and preclude robust sex-specific analyses. Future prospective studies with larger, more diverse populations, comprehensive cardiac and coronary assessments, sufficient clinical outcomes, and prespecified sample size calculations are warranted to validate these findings and clarify whether NT-proBNP contributes to endothelial dysfunction or primarily reflects shared upstream cardiac–renal–vascular pathophysiology in patients with CAD.

## 5 Conclusion

In patients with angiographically confirmed CAD, circulating NT-pro-BNP levels were independently associated with impaired endothelial function as assessed via DTM. High NT-pro-BNP concentrations were associated with low VRI values and vascular reactivity dysfunction. Based on these findings, NT-pro-BNP, beyond its established role as a biomarker of myocardial wall stress, may also reflect systemic vascular impairment in patients with CAD. Although causality cannot be inferred from this cross-sectional analysis, NT-pro-BNP levels may be an integrative marker linking cardiac load and endothelial dysfunction. Prospective studies must be performed to determine whether incorporating NT-pro-BNP levels into vascular risk assessment strategies improves cardiovascular risk stratification and clinical outcomes.

## Data Availability

The original contributions presented in the study are included in the article/Supplementary Material, further inquiries can be directed to the corresponding authors.
